# Supplementary material for: Tunable templating of photonic microparticles via liquid crystal order-guided adsorption of amphiphilic polymers in emulsions
Source: Nat Commun. 2024 Feb 15;15:1404. doi: 10.1038/s41467-024-45674-5 (PMC10869789; doi:10.1038/s41467-024-45674-5)
Supplement: Supplementary file 1 — Supplementary information [file 41467_2024_45674_MOESM1_ESM.pdf]

Supplementary information for
Tunable templating of photonic microparticles via liquid crystal order-guided adsorption of amphiphilic polymers in emulsions

by

Xu Ma, Yucen Han, Yan-Song Zhang, Yong Geng, Apala Majumdar and Jan P.F. Lagerwall

Supplementary Methods

Microfluidic device assembly and shell production

Shell production with the microfluidic device is shown in Supplementary Fig. 1. Two cylindrical borosilicate glass capillaries (inner and outer diameter 0.5 and 1 mm, respectively, Science Products GmbH) were first tapered by a micropipette puller (P-100, Sutter Instruments) and then cut using a Micro Forge (Narishige, MF-900), creating orifices with inner diameters around 120 and 210 μm for the injection and collection capillaries, respectively. The injection capillary was treated to give it a hydrophobic surface character by immersing it into a 1 wt% aqueous solution of octadecyldimethyl (3-trimethoxysilylpropyl) ammonium chloride (DMOAP, Sigma-Aldrich) for 5 mins. Afterward it was flushed by distilled water

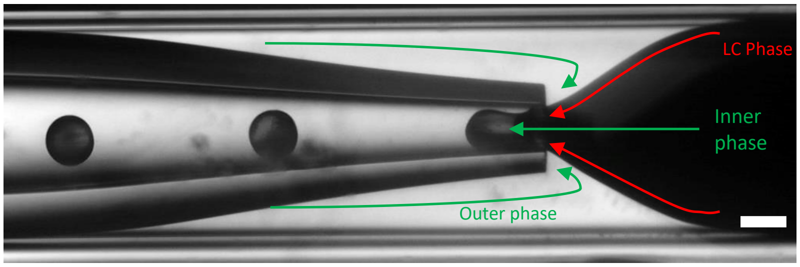

Supplementary Fig. 1: **Shell production using a glass capillary microfluidic set-up.** The production was done around 10°C below the clearing temperature for each liquid crystal (LC) mixture. Arrows represent flow directions of each phase; red for the LC phase and green for the aqueous phases. For mixtures with low HDDA content, the heating is important to reduce the effective viscosity. Scale bar: 200μm.

and then dried in an oven at 80°C for about 12h. The injection and collection capillaries were inserted head to head into a 2 cm long square cross section capillary with 1 mm inner side length (CM Scientific).

Characterization of CLC mixtures as a function of HDDA addition

Samples of each of the seven CLC mixtures (components in Supplementary Fig. 2, compositions in Supplementary Table 1) were investigated with respect to their phase sequences by DSC, see Supplementary Fig. 3. For each mixture with HDDA, around 3–10 mg was placed in a crimped DSC aluminum pan, rapidly cooled to −40°C, and then a program with subsequent heating to 90°C at 5°C/min, cooling back to −40°C at 5°C/min and then reheating to 90°C at 10°C/min, was carried out under nitrogen environment. As shown in Supplementary Fig. 3a, components of the CLC base mixture, without HDDA, crystallized during the first rapid cooling to −40°C, in contrast to the case with HDDA-containing mixtures.

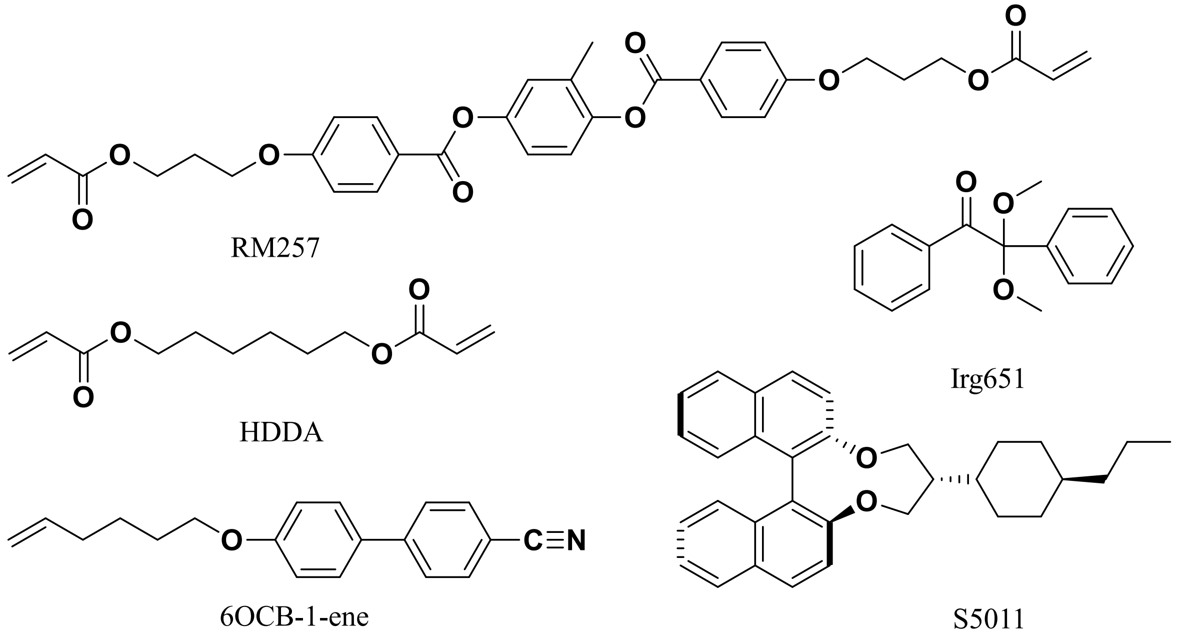

Supplementary Fig. 2: **Chemical structures of monomers used to prepare CLC mixtures.** The abbreviations used for each molecule are defined in the Methods section of the main paper, where also the full IUPAC names are provided.

Supplementary Table 1: Compositions of CLC mixtures. All numbers are wt.-%.

Retroreflection	HDDA	6OCB-1-ene	RM257	S5011	Irg651
Red	0	50	46	2	2
Red	3	50	43	2	2
Red	6	50	40	2	2
Red	9	50	37	2	2
Red	10	50	36	2	2
Red	11	50	35	2	2
IR	6	50	40.8	1.2	2

Each mixture was also investigated with respect to its effective shear viscosity at 23°C, using a microfluidic flow viscosimeter operating at a flow rate of 0.5 μL/min. Each sample had a volume of 1 mL. Given that a nematic liquid crystal has five independent viscosity parameters, the read-out of the device must be interpreted with care. We do not wish to attribute the measured value to any of the actual liquid crystal viscosities, but rather consider it an abstract benchmark for comparing effective shear viscosities within the series of mixtures considered. Since the mixtures are subject to a similar shear flow during microfluidic shell production, we consider these data useful with respect to optimization of the mixtures for

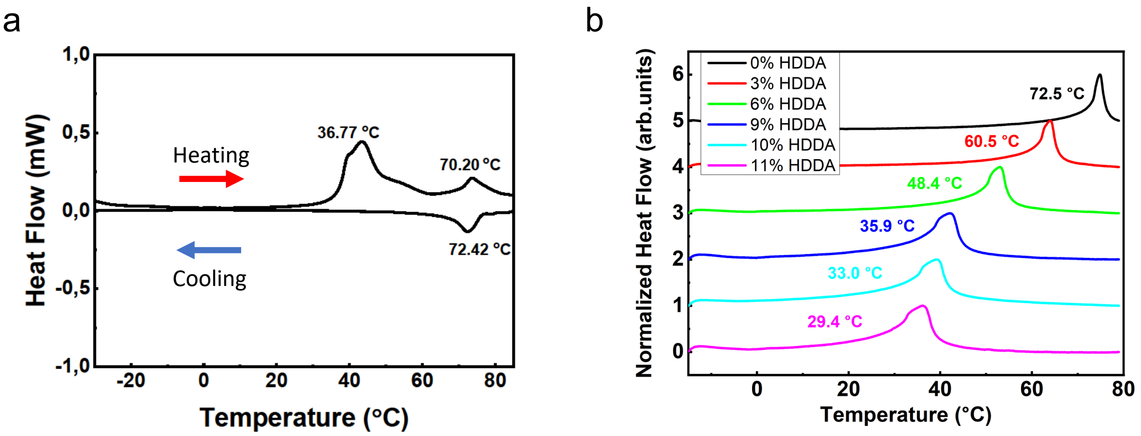

Supplementary Fig. 3: **Calorimetric characterization of different precursor mixtures.** HDDA=1,6-hexanediol diacrylate. (a) Differential scanning calorimetry (DSC) thermograms (first heating run, with a rate of 5°C/min.) of the crystallized CLC base mixture, without HDDA. The indicated temperatures are onset melting and clearing temperatures. (b) DSC thermograms (second heating run, with a rate of 10°C/min.) of CLC mixtures with different HDDA concentrations. The indicated temperatures are the onset temperatures for clearing in each mixture. Source data are provided as a Source Data file.

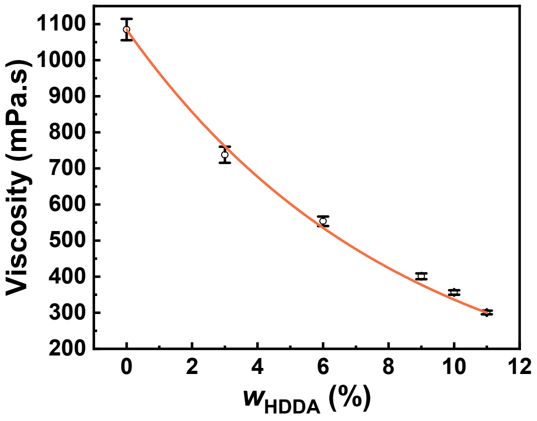

Supplementary Fig. 4: **Impact of varying content of 1,6-hexanediol diacrylate (HDDA) in precursor mixtures on their viscosity.** The effective shear viscosity (see text for comments on interpretation) at room temperature of CLC mixtures as a function of w_{HDDA}, i.e., the mass percentage of HDDA in the CLC mixture. The error bars represent standard error ($S.E.$) obtained by following the formula $SE = \frac{\delta}{\sqrt{N}}$, where δ is sample standard deviation and N is number of samples and here for all data point $N = 3$. For each point, from left to right, $S.E. = 29.5$, 22.4, 13.4, 8.0, 6.3 and 5.3 mPa s, respectively. Source data are provided as a Source Data file.

making shells. The results, shown in Supplementary Fig. 4, suggest that this effective shear viscosity decays exponentially with increasing HDDA content.

Calibration of sample temperature when working with open hot stage

During the experiment in Fig. 4 in the main paper, the shells are in a droplet of the PVA solution on a glass slide, without any cover glass, thus with a top interface to air. This means that water evaporates as the sample is heated, and if the hot stage lid is closed, the water condenses on the top glass, blurring the images. For this reason we work with open hot stage, which means that the reported temperatures are higher than the actual sample temperatures. In Supplementary Fig. 5 we show reference experiments where we monitor the actual sample temperature during open hot stage operation using a thermal imaging camera (Infratech, Germany). Within the relevant temperature window, we find an approximately

linear calibration curve which shows that the actual sample temperature when transitions are observed in Fig. 4 corresponds very well to those of other experiments conducted with closed hot stage. At an open hot stage setting of 50°C, corresponding to the setting where we see the alignment transition in Fig. 4, the sample has an actual temperature of 44.5°C. This is very similar to the temperature of the alignment transition found with closed hot stage in Supplementary Fig. 6, hence we can safely conclude that the discrepancy in the nominal temperature is due to the operation with open hot stage.

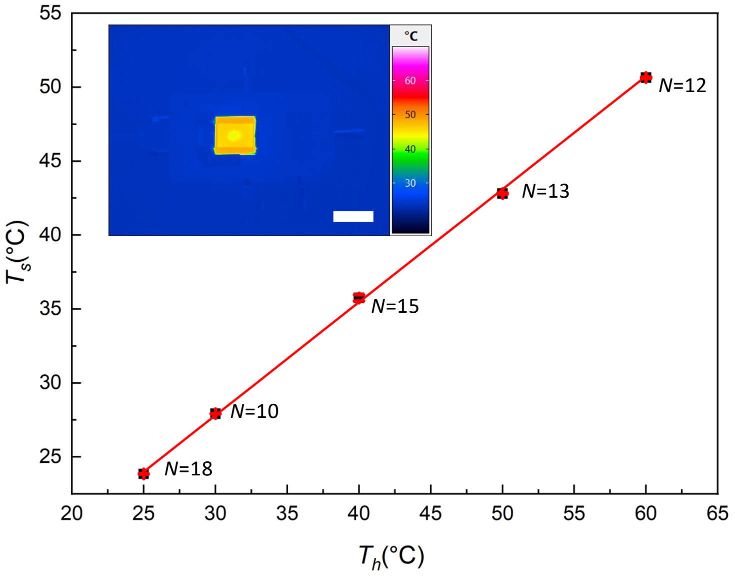

Supplementary Fig. 5: **Correlation between set and real sample temperature during operation with open hot stage.** The temperature T_s of a drop of an aqueous CLC shell suspension on a glass slide in the open hot stage, placed in the viewing area as during microscopy, as a function of target temperature T_h of the hot stage controller. The variable N represents the number of measurements taken to establish each data point. The error bars represent standard error ($S.E.$) obtained by following the formula: $SE = \frac{\delta}{\sqrt{N}}$ where δ is the sample standard deviation. $S.E.$ is 0.01, 0.01, 0.21, 0.05 and 0.05°C from left to right. As a representative example, the inset shows a thermal imaging camera photo of the sample when the hot stage controller is set to 50°C. Scale bar: 40mm. The color legend defines the temperature representation. Source data are provided as a Source Data file.

Mathematical Modeling of Director Fields for Different Boundary Conditions

The Free Energy

In the LdG framework, the ordering of a CLC sample is described by a macroscopic order parameter: the LdG **Q**-tensor order parameter, which is a symmetric, traceless 3×3 matrix.[1]

$$\mathbf{Q} = \begin{pmatrix} q_1 & q_2 & q_3 \\ q_2 & q_4 & q_5 \\ q_3 & q_5 & -q_1 - q_4. \end{pmatrix} \tag{1}$$

The eigenvectors of the **Q**-tensor model the preferred directions of averaged molecular alignment, and the eigenvalues are a measure of the degree of orientational order about the corresponding eigenvectors. The director, **n**, is defined to be the eigenvector with the largest positive eigenvalue. A CLC phase is said to be isotropic if $\mathbf{Q} = 0$, uniaxial if the LdG **Q**-tensor has a pair of equal non-zero eigenvalues and the director is the eigenvector with the non-degenerate eigenvalue, and the CLC phase is biaxial if the associated **Q**-tensor has three distinct eigenvalues. The LdG theory is a variational theory and the equilibrium or physically observable configurations in experiments are modelled by local or global energy minimisers, subject to the imposed boundary conditions. Structural transitions and defects are often associated with biaxiality, and in what follows, we only focus on the director of the numerically computed energy minimisers, and track structural transitions in the director of the energy minimisers as a function of the imposed boundary conditions on the shell surfaces.

The LdG free energy for CLCs is given by[2]

$$F(\mathbf{Q}) = \int_{\Omega} \frac{K_0}{2}(\nabla \cdot \mathbf{Q})^2 + \frac{K_1}{2}|\nabla \times \mathbf{Q} + 2q_0\mathbf{Q}|^2 + f_b(\mathbf{Q})dV, \tag{2}$$

where the bulk energy density is given by

$$f_b(\mathbf{Q}) := \frac{A}{2}\mathrm{tr}\mathbf{Q}^2 - \frac{B}{3}\mathrm{tr}\mathbf{Q}^3 + \frac{C}{4}(\mathrm{tr}\mathbf{Q}^2)^2. \quad (3)$$

Here K_0 and K_1 are material-dependent elastic constants, usually associated with characteristic splay and twist deformations, and $q_0 = 2\pi/p_0$. For an achiral nematic, $q_0 = 0$. A is a temperature dependent constant, i.e., $A = \alpha(T - T^*)$ where α is a positive material-dependent constant and T^* is a critical material-dependent temperature, B and C are positive material dependent constants. The minimisers of f_b dictate the nematic ordering in spatially homogeneous samples, and their dependence on the temperature. The minimisers of f_b are isotropic for high temperatures ($A > \frac{B^2}{27C}$) and the minimisers of f_b are ordered uniaxial states for low temperatures defined by $A < 0$. We note, $|\,.\,|$ is the matrix norm, so that

$$|\mathbf{Q}| = \sqrt{\mathrm{tr}\mathbf{Q}^2} = \sqrt{Q_{ij}Q_{ij}}. \quad (4)$$

Using summation notation, we have $\nabla \cdot \mathbf{Q} = \partial_\alpha Q_{i\alpha}$ and $\nabla \times \mathbf{Q} = \epsilon_{i,j,k}\partial_j Q_{k\beta}$, where $\epsilon_{i,j,k}$ is the Levi-Civita antisymmetric symbol, and $i, j, k = 1, 2, 3$. The equilibrium or the physically observable configurations are either local or global minimisers of (2) subject to the imposed boundary conditions.

Our domain is the spherical shell defined by, $\Omega = B(\mathbf{0}, R_o)\backslash B(\mathbf{x}_c, R_i)$, where $B(\mathbf{x}_c, R_i) \subset B(\mathbf{0}, R_o)$. The choice of $\mathbf{x}_c$ differentiates between symmetric and asymmetric shells i.e. symmetric shells have $\mathbf{x}_c = \mathbf{0}$ i.e. the origin in three dimensions, and R_i and R_o are the radii of the inner and outer shells. We nondimensionalize the system using the following rescaling

$$\bar{\mathbf{x}} = \mathbf{x}/R_o,\ \bar{\mathbf{Q}} = \sqrt{\frac{27C^2}{2B^2}}\mathbf{Q},\ \bar{\mathcal{F}} = \frac{27C^3}{2B^4R_o^3}\mathcal{F}. \quad (5)$$

Dropping all bars for convenience, the dimensionless LdG energy can be written as

$$\mathcal{F}(\mathbf{Q}) = \int_\Omega \left\{ \frac{\xi_R^2}{2}((\nabla \cdot \mathbf{Q})^2 + \eta \left|\nabla \times \mathbf{Q} + 2\sigma \mathbf{Q}\right|^2) + \frac{t}{2}\mathrm{tr}\mathbf{Q}^2 - \sqrt{6}\mathrm{tr}\mathbf{Q}^3 + \frac{1}{2}(\mathrm{tr}\mathbf{Q}^2)^2 \right\} d\mathbf{x}. \quad (6)$$

The nondimensionalised domain, $\Omega = B(\mathbf{0}, 1)\backslash B(\mathbf{x}_c/R_o, \rho)$, $\rho_c = R_i/R_o$, where $\mathbf{x}_c/R_o = (0, 0, c)$, c and ρ satisfy $c > \rho > 0$ and $c + \rho < 1$. If $c = 0$, we have a symmetric shell. The parameter $t = \frac{27AC}{B^2}$ is the reduced temperature, $\xi_R = \sqrt{\frac{27CL_1}{B^2R_o^2}}$, $\eta = \frac{K_1}{K_0}$, is a measure of the elastic anisotropy and equal elastic constants correspond to $\eta = 1$; the parameter $\sigma = q_0 R_o$, where q_0 has been defined above.

The inner and outer shells surfaces are denoted by $\partial\Omega_i$ and $\partial\Omega_o$. We perform multiple numerical computations by imposing either tangential or normal boundary conditions on the inner and outer surfaces, or hybrid boundary conditions. For both surfaces, the boundary conditions are imposed by means of surface anchoring energies. There is considerable freedom in the choice of the surface energies and the anchoring coefficients, and our results are by no means comprehensive, but do serve as good numerical illustrations.

Normal boundary conditions require the director to be normal or orthogonal to the shell surface. Such anchoring can be imposed on either the inner or outer shell surfaces (or both) by means of the following surface energy[3]

$$F_s = \frac{\omega}{2} \int_{\partial\Omega_k} |\mathbf{Q} - \mathbf{Q}^{\perp}|^2 dA, \ k = i \ or \ o, \quad (7)$$

where ω is the reduced anchoring strength and

$$\mathbf{Q}^{\perp} = s_+(\mathbf{v} \otimes \mathbf{v} - \frac{\mathbf{I}}{3}), \quad (8)$$

where $\mathbf{v}$ is the surface normal of $\partial\omega_i$. If ω is small, then the anchoring is weakly imposed, and if ω is large, then the anchoring is strongly imposed. We work with fixed values of ω, but in practice, ω is dynamically tuned in experiments by varying temperature.

The tangential boundary conditions are enforced by the following surface energy,

$$F_s = \int_{\partial\Omega_k} \frac{\omega_1}{2}|\tilde{\mathbf{Q}} - \tilde{\mathbf{Q}}^{\|}|^2 + \frac{\omega_2}{2}(tr\tilde{\mathbf{Q}}^2 - s_+^2)^2 dA,\ k = i\ or\ o, \tag{9}$$

where

$$\tilde{\mathbf{Q}} = \mathbf{Q} + \frac{\mathbf{s_+ I}}{3},\ \tilde{\mathbf{Q}}^{\|} = \mathbf{P}\tilde{\mathbf{Q}}\mathbf{P},\ \mathbf{P} = \mathbf{I} - \mathbf{v} \otimes \mathbf{v}, \tag{10}$$

$\tilde{\mathbf{Q}}^{\|}$ is the tangential projection of $\mathbf{Q}$ on the shell surfaces, $\mathbf{v}$ is the normal to the shell surface, ω_1 is the reduced anchoring strength that favors the tangential boundary conditions or favours the director, $\mathbf{n}$, to be tangent to the shell surfaces and ω_2 is an anchoring coefficient that enforces the preferred degree of orientational ordering on the shell surfaces.

Numerical Method

Following the methods in [4] , we numerically model the domain Ω using the bispherical coordinate system, (ξ, μ, ψ), which is also given by[5]

$$x = \frac{a \sin\mu \cos\psi}{\cosh\xi - \cos\mu},\ y = \frac{a \sin\mu \sin\psi}{\cosh\xi - \cos\mu},\ z = \frac{a \sinh\xi}{\cosh\xi - \cos\mu} \tag{11}$$

where (x, y, z) are Cartesian coordinates,

$$a = \frac{\sqrt{(1 - \rho^2 - c^2)^2 - 4c^2\rho^2}}{2c}. \tag{12}$$

For fixed ξ, (μ, ψ) represent a spherical surface given by

$$x^2 + y^2 + (z - a\coth\xi)^2 = \frac{a^2}{\sinh^2\xi}. \tag{13}$$

The shell surfaces, $\partial\Omega_i$ and $\partial\Omega_o$, correspond to ξ_i and ξ_o, where

$$\xi_o = \sinh^{-1}(a),\ \xi_i = \sinh^{-1}(a/c). \tag{14}$$

Let $\zeta = 2(\xi - \xi_o)/(\xi_i - \xi_o) - 1$ and the original domain is mapped to

$$\Omega = \{(\zeta, \mu, \psi) | -1 \leq \zeta \leq 1, 0 \leq \mu \leq \pi, 0 \leq \psi \leq 2\pi\}. \tag{15}$$

We expand the tensor function $\mathbf{Q}$ in terms of real spherical harmonics of (μ, ψ) and Legendre polynomials of ζ,

$$q_i(\zeta, \mu, \psi) = \sum_{l=0}^{L-1} \sum_{m=1-M}^{M-1} \sum_{n=|m|}^{N-1} A_{lnm}^{(i)} Z_{lnm}(\zeta, \mu, \psi), \tag{16}$$

where $N \geq M \geq L \geq 0$ specify the truncation limits of the expanded series, with

$$Z_{lnm}(\zeta, \mu, \psi) = P^l(\zeta) Y_{nm}(\mu, \psi), \tag{17}$$

$$Y_{nm} = P_n^{|m|}(\cos\mu) X_m(\psi), \tag{18}$$

$$X_m(\psi) = \begin{cases} \cos m\psi, & \text{if } m \geq 0, \\ \sin |m|\psi, & \text{if } m < 0. \end{cases} \tag{19}$$

and $P_n^m(x)$ $(m \geq 0)$ are the normalized associated Legendre polynomials. Using this series expansion, the LdG energy of $q_i,\ i = 1, \cdots, 5$ is a function for the $5NML$ unknowns. Substituting (16) into the non-dimensionalized free energy (6) and surface energy (7) and/or (9), we obtain a free energy as a function of these unknown tensor order parameter elements, $A_{lnm}^{(i)}$. The redefined free energy function is then minimized by using a standard optimization method, such as L-BFGS[6] that treats the independent elements of tensor $A_{lnm}^{(i)}$ as variables. Most of simulation results in this paper are obtained by taking $(N, L, M) = (64, 32, 64)$.

Supplementary Notes

Shells of HDDA-doped CLC mixtures

Supplementary Fig. 6 shows the transmission POM textures of a shell with 6% HDDA stabilized between inner and outer isotropic phases of the standard aqueous PVA 2 solution, as it is heated from room temperature to about 49°C, at which the CLC mixture is completely isotropic. We recognize the exact same qualitative texture development from tangential (a–d) to FCD configuration (e–k) as for the HDDA-free CLC shell stabilized with the same PVA solution in Fig. 2 in the main paper, but the temperatures of realignment are depressed just as the clearing temperature is depressed in this mixture compared to the base mixture. At the beginning, the CLC phase is tangentially aligned (Supplementary Fig. 6a-d) but at $T_t = 45.8$°C, a major rearrangement toward FCDs is seen near the bottom of the shell (Supplementary Fig. 6e-l). The FCD texture persists and intensifies as the temperature increases, eventually filling the sphere surface at about 47°C, see Supplementary Fig. 6h-j. Clearing starts at $T_{N^*I} \approx 48.5$°C, with only the last traces of FCDs being visible at 48.7°C (m) before the shell is fully isotropic at 48.9°C (n). If the HDDA content is increased to 10% or more, the FCD texture is present already at room temperature, as shown in Supplementary Fig. 7.

To probe the impact of average shell thickness, we prepare three sets of shells, each with a different thickness but about the same external radius, see Supplementary Fig. 8. The temperature where realignment into FCD configuration starts is the lowest in the thinnest shells and increases consistently with increasing average shell thickness. In this experiment we have a slightly higher clearing point than in the other experiments with the 6% HDDA CLC mixture, reflecting the difficulty in reproducing the exact same mixture composition due to the many components. This does not affect the qualitative conclusions.

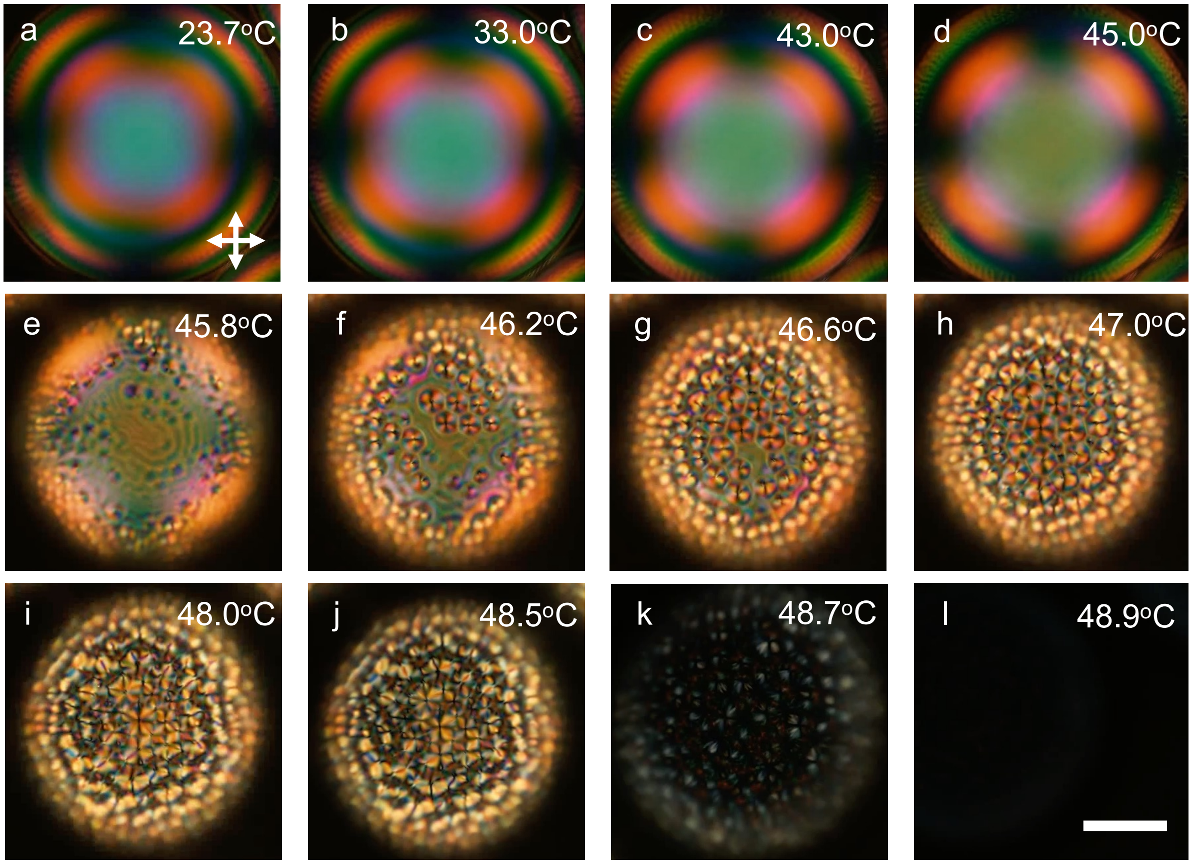

Supplementary Fig. 6: **Alignment transition upon heating a shell of cholesteric liquid crystal (CLC) containing 6% 1,6-hexanediol diacrylate (HDDA) stabilized by polyvinylalcohol (PVA).** Transmission POM images (polarizer orientation indicated in (a)) of a CLC shell produced with a 6 wt.% HDDA mixture and stabilized by our standard PVA solution as it is heated from room temperature to the isotropic state. The heating rate was separated in three ranges: 5°C/min for 23.7 – 43°C, 1°C/min for 43 – 45°C, and 0.3°C/min for 45 – 50°C. The focus is at the shell equator in (a-d) and at the bottom surface in (e-l). Scale bar: 50μm.

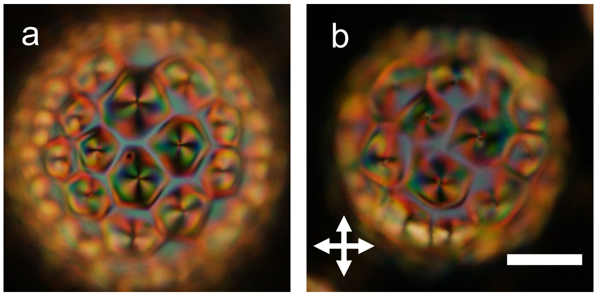

Supplementary Fig. 7: **Cholesteric liquid crystal (CLC) shells with focal conic domain (FCD) configuration at room temperature.** Transmission POM images (polarizer orientation indicated in (b)) of CLC shells produced with 10 wt.% (a) and 11 wt.% (b) 1,6-hexanediol diacrylate (HDDA), stabilized by our standard polyvinylalcohol (PVA 2), after annealing at room temperature. Scale bar: 50μm.

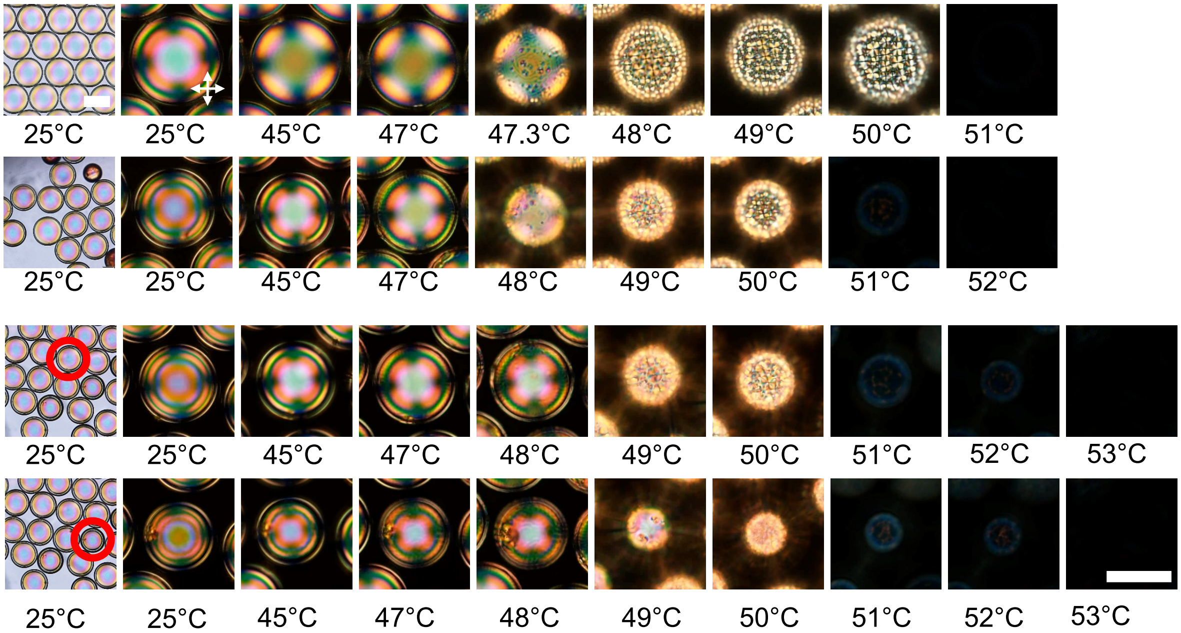

Supplementary Fig. 8: **Test of impact of shell thickness on alignment transition.** Three sets of shells of the CLC mixture with 6 wt.% HDDA stabilized by 5 wt.% PVA 2 in water, produced using a Secoya Raydrop emulsification set-up, yielding shells with radius about 100 μm. The two bottom rows focus on two different shells in the same set, as indicated by the red rings in the left column. The shells in the three sets differ in their average thickness, which leads to slightly different apparent temperature of the alignment change. Polarizer indications indicated in top row, second column. Scale bar: 100μm.

F-127-stabilized CLC shells

When stabilizing a shell of the 6% HDDA CLC mixture by F-127, the FCD configuration is found already at room temperature, as shown in Supplementary Fig. 9a. By cooling the sample in a fridge to 5°C, we see the reverse transition compared to what was studied in the rest of the paper, with the tangential texture developing out of the FCD texture as the shell is cooled far from the clearing point of the mixture, see Supplementary Fig. 9b.

If we instead use the base CLC mixture, without any HDDA, for shells stabilized by F-127, the texture is tangential at room temperature and transforms to FCD configuration upon heating, as shown in Supplementary Fig. 10. The behavior is qualitatively similar to the corresponding experiment with PVA stabilization in Fig. 2 in the main paper, but the onset temperature of the realignment (Supplementary Fig. 10c) is much lower when F-127 is used for stabilizing the shell.

To gain a different view of the alignment transition we also repeat this experiment with the microscope tilted by 90°, such that the shells are observed perpendicular to gravity. This allows to study the textural change at all different thicknesses within a shell simultaneously, since the thickest point is now at the bottom and the thinnest point is at the top in the image,

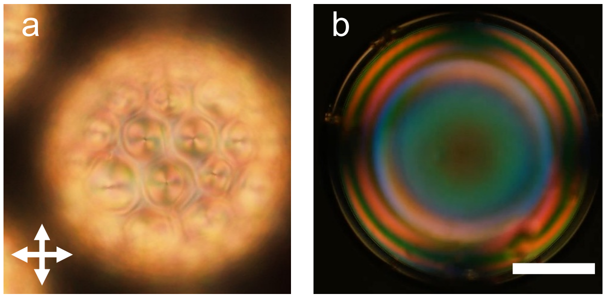

Supplementary Fig. 9: **Alignment transition upon cooling a shell of cholesteric liquid crystal (CLC) containing 6% 1,6-hexanediol diacrylate (HDDA) stabilized by the surfactant F-127.** Transmission POM images (polarizer orientation indicated in (a)) of a CLC shell produced with a 6 wt.% HDDA mixture stabilized by F-127 after annealing at room temperature. The texture at room temperature is shown in (a), while (b) shows the texture after placing the shell in a fridge at 5°C overnight. Scale bar: 50μm.

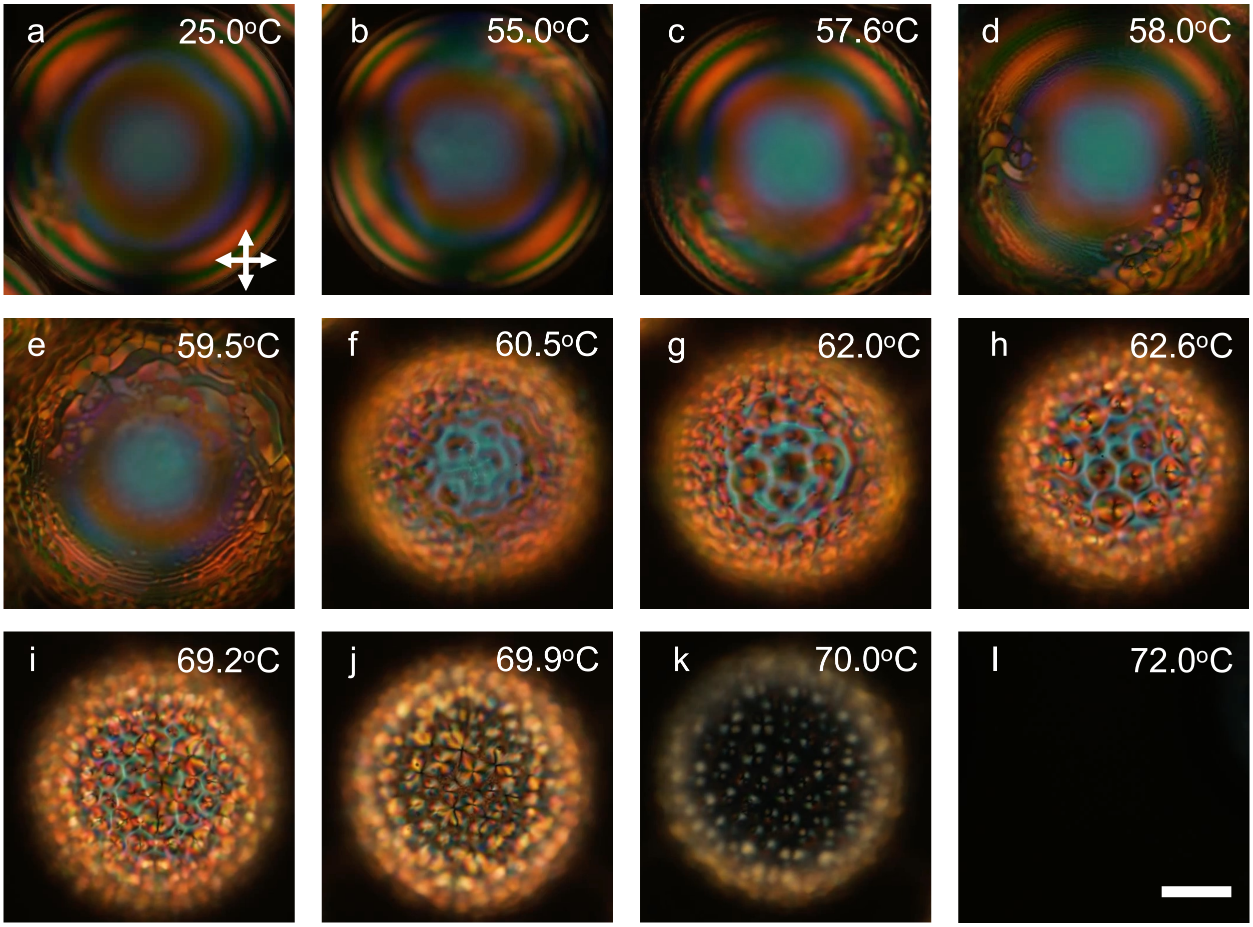

Supplementary Fig. 10: **Alignment transition upon heating a shell of cholesteric liquid crystal (CLC) without 1,6-hexanediol diacrylate (HDDA) stabilized by the surfactant F-127.** Transmission POM images (polarizer orientations indicated in (a)) of a CLC shell produced with a 0 wt.% HDDA mixture and stabilized by 1 wt.% aqueous solution of Pluronic F-127 block copolymer surfactant as inner and outer phase, as it is heated from room temperature to isotropic. The heating rate was separated in two ranges: 5°C/min for 23.5 – 55°C and 1°C/min for 55 – 72°C. The focus is at equator of the shell in (a-e) and at the bottom surface in (f-l). Scale bar: 50μm.

see Supplementary Fig. 11 and Supporting Movie 8. As T_t is reached, we first see a periodic set of parallel lines developing along the thickness gradient (Supplementary Fig. 11d–f). Slightly later in the heating process these lines get laterally modulated, and then the FCDs develop from the increasingly modulated lines.

This development resembles very much the development of toric focal conic domains following a nematic-SmA transition in hybrid alignment, as initially studied by Cladis and

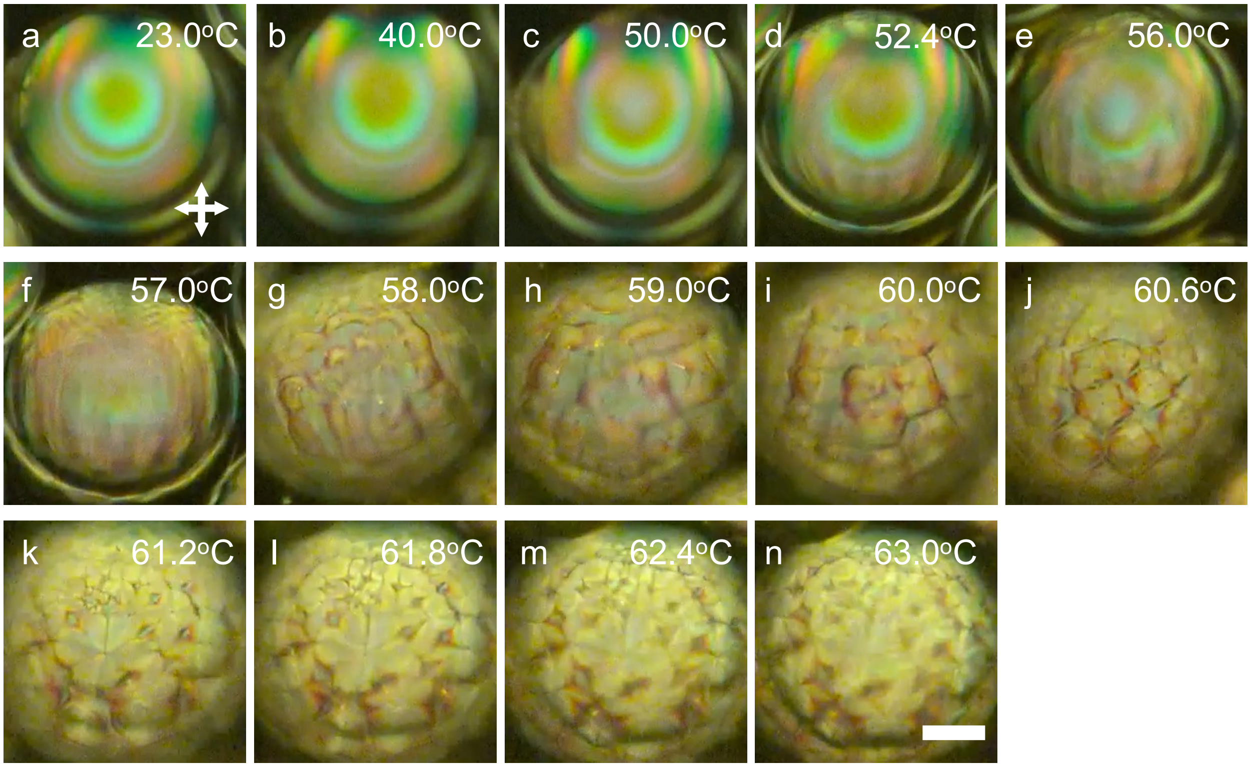

Supplementary Fig. 11: **Alignment transition upon heating a shell of cholesteric liquid crystal (CLC) without 1,6-hexanediol diacrylate (HDDA) stabilized by the surfactant F-127, observed perpendicular to gravity.** Transmission POM images (polarizer orientations indicated in (a)) from the side (obtained by tilting the microscope by 90° with green filter on the light source) of CLC shells produced with a 0 wt.% HDDA mixture and stabilized by 1 wt.% aqueous solution of Pluronic F-127 block copolymer surfactant as inner and outer phase, as the suspension is heated from room temperature to 63°C. The heating rate was separated in five ranges: 5°C/min for 23 – 40°C, 1°C/min for 40 – 50°C, $0,3$°C/min for $50-56$°C, $0,1$°C/min for $56-60$°C and $0,01$°C/min for $60-63$°C. The focus is at the equator of the shells in (a-f) and at the front surface in (g-n). Scale bar: 50μm.

Torza for flat films,[7] the corresponding experiment in shells studied by us.[8,9] While in those cases the transition is driven by a phase transition with permanent boundary conditions, in the present case it is instead driven by a change of effective boundary conditions while the thermodynamic phase remains the same. In some shells the thin region is so thin that FCD formation is suppressed (see Supplementary Movie 6) but in most shells in the experiment the entire shell gets covered with FCDs. The FCD size increases with thickness of the shell, confirming in the liquid crystal state our conclusions drawn from the polymerized solid shells in Fig. 3d-e in the main paper.

We also conduct a series of experiments with the same combination of shell composition and stabilizer where we stop the heating before the isotropic state is reached, cooling the sample back to room temperature, see Supplementary Fig. 12. In all cases the realignment is confirmed to be fully reversible, as the tangential alignment is fully recovered around room temperature. However, it is quite rich in defects immediately after recooling, hence annealing is required to obtain uniform optical properties.

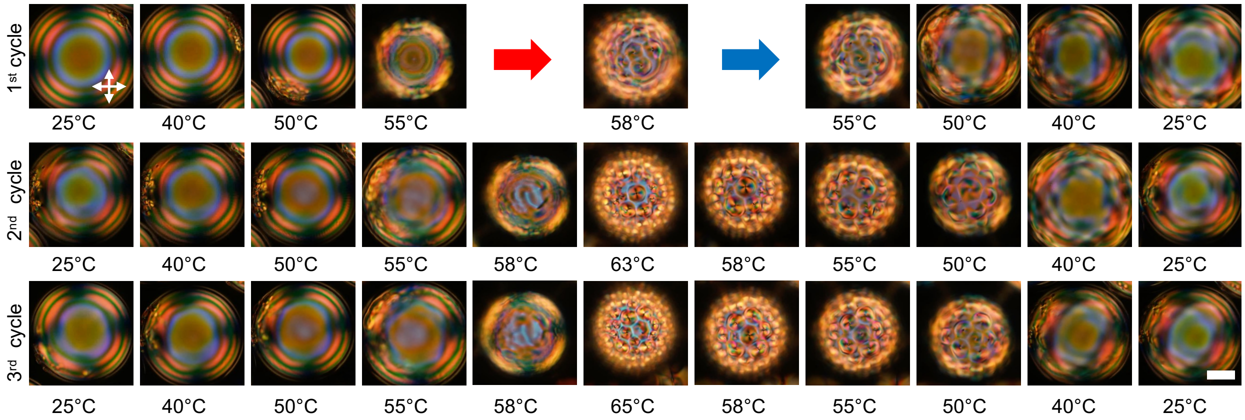

Supplementary Fig. 12: **Test of dynamics and reversability of alignment transition.** Transmission POM images (polarizer orientation indicated in first image) of a CLC shell produced with a 0 wt.% HDDA mixture and stabilized by 1 wt.% F-127 as it is heated from room temperature to the focal conic state and then cooled back to room temperature. The heating and cooling rate was different in three different cycles. For the first cycle: 5°C/min for 24 – 40°C, 1°C/min for 40 – 50°C, 0.1°C/min for 50 – 58°C, 0.1°C/min for 58 – 50°C, 1°C/min for 50 – 40°C, 5°C/min for 40 – 25°C. For the second cycle: 5°C/min for 25 – 40°C, 1°C/min for 40 – 50°C, 0.5°C/min for 50 – 63°C, 0.5°C/min for 63 – 50°C, 1°C/min for 50 – 40°C, 5°C/min for 40 – 25°C. For the third cycle: 5°C/min for 25 – 40°C, 1°C/min for 40 – 65°C, 1°C/min for 65 – 40°C, 5°C/min for 40 – 25°C. There are 10 minutes interval between two cycles. Scale bar: 50μm.

Comparison between different PVA qualities and Pluronic types as stabilizer

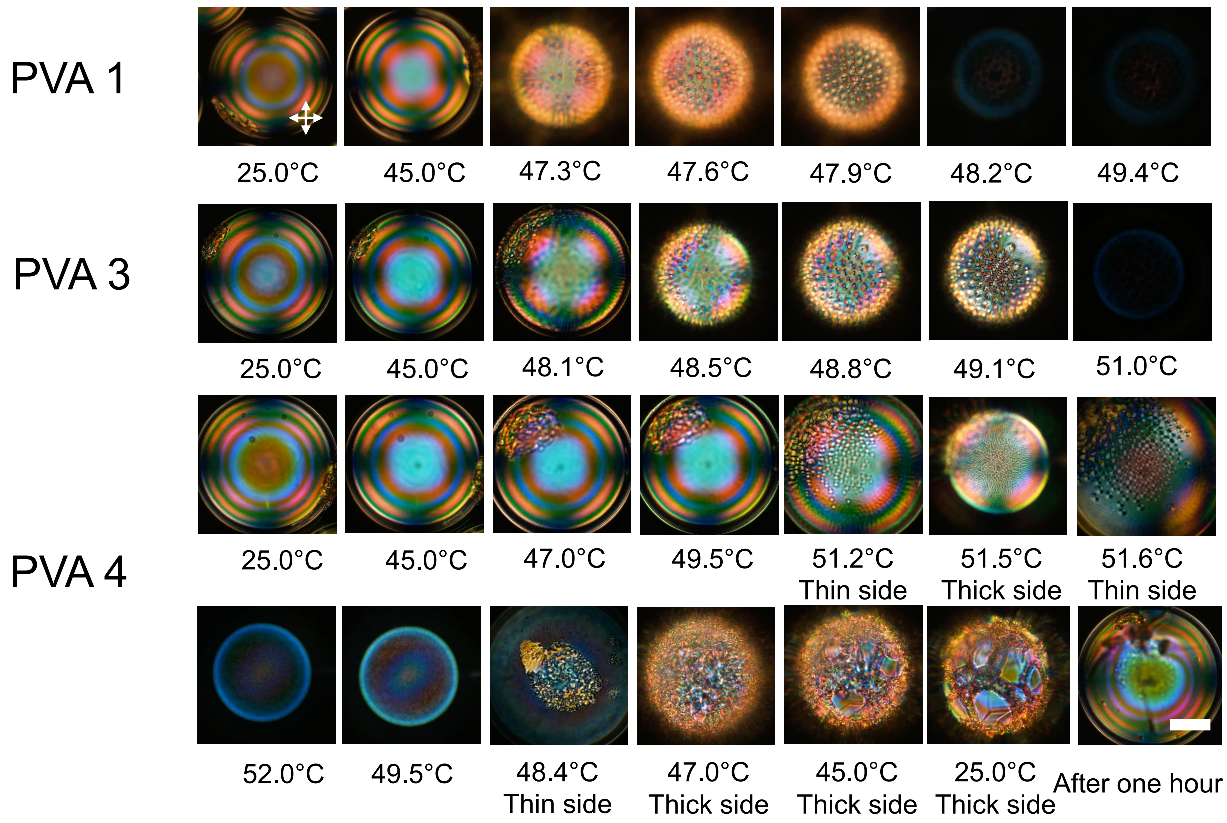

Supplementary Fig. 13: **Comparison of different types of polyvinylalcohol (PVA) used as stabilizer.** Transmission POM images (polarizer orientation indicated in first image) of realignment upon heating of a shell with 6 wt.% HDDA stabilized by (row 1) PVA 1 (80% hydrolysis), (row 2) PVA 3 (85–87% hydrolysis, high molar mass) and (rows 3–4) PVA 4 (98% hydrolysis). Shells stabilized by PVA 4 remain tangential-aligned almost all the way to the clearing transition on the thick side, but there are some FCDs appearing on the thin side. In contrast to shells stabilized by amphiphilic polymers, the shell survives being heated into the isotropic phase (row 3) and then cooled back to cholesteric (row 4), the shell recovering a good tangential alignment after annealing. Scale bar: 50μm.

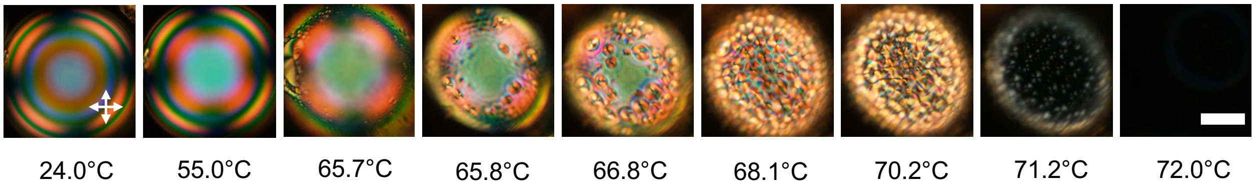

Supplementary Fig. 14: **Alignment transition upon heating a shell of cholesteric liquid crystal (CLC) without 1,6-hexanediol diacrylate (HDDA) stabilized by the surfactant F-108.** Transmission POM images (polarizer orientation indicated in first image) of realignment upon heating of a shell with 0 wt.% HDDA stabilized by Pluronic F-108. Scale bar: 50μm.

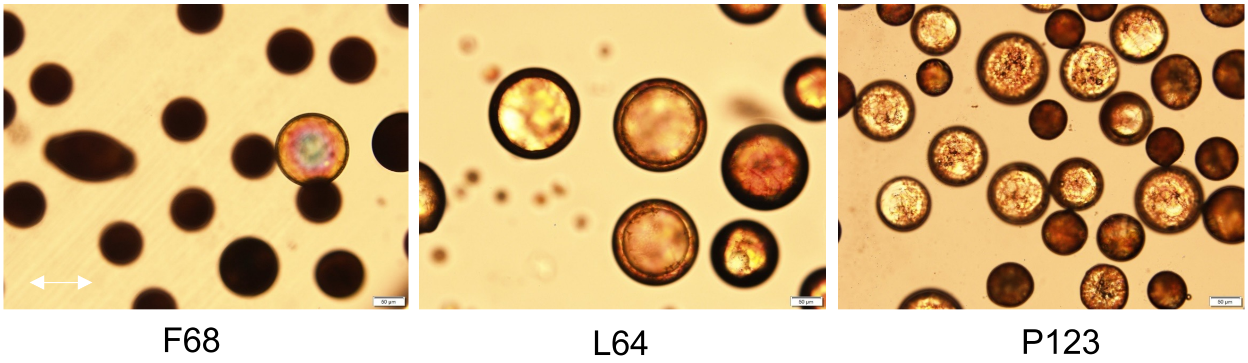

Supplementary Fig. 15: **Results of attempts of producing shells of cholesteric liquid crystal (CLC) stabilized by inappropriate Pluronic surfactants.** Textures of shells or droplets produced with Pluronic F-68, L-64 and P-123, respectively, as stabilizer, none of them providing acceptable shell stability. Scale bar: 50μm.

Numerical results on symmetric shells

All results from numerical simulation of asymmetric shells are shown in the main paper. Our numerical results for symmetric shells are shown in Figures 16 and 17.

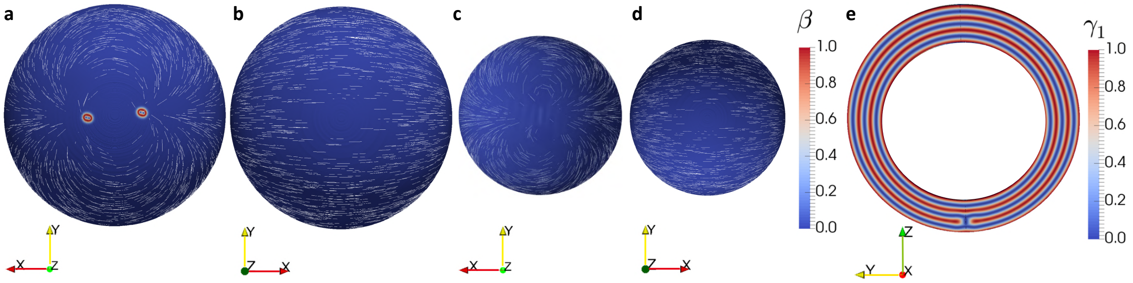

Supplementary Fig. 16: **Simulated configuration of symmetric cholesteric liquid crystal (CLC) shell with tangential boundary conditions.** An equilibrium profile on a symmetric shell with tangential boundary conditions on the inner and outer surfaces, at fixed temperature $t = -1.79$, $\xi_R = 1/50$, $\eta = 1$, $\sigma = 10\pi$, $\omega_1 = \omega_2 = 0.1$. (a) Bottom of outer surface; (b) top of outer surface; (c) bottom of inner surface; (d) top of inner surface; (e) cross-section. The colorbars label the $\beta = 1 - 6\frac{\left(tr\mathbf{Q}^3\right)^2}{\left(tr\mathbf{Q}^2\right)^3}$ in (a-d), $\gamma_1 = |\mathbf{n} \cdot \mathbf{e}_x|$ in (e). The white lines in (a-d) label the director $\mathbf{n}$, which is the eigenvector of $\mathbf{Q}$ with the largest eigenvalue. The red dots in (a) signify topological defects.

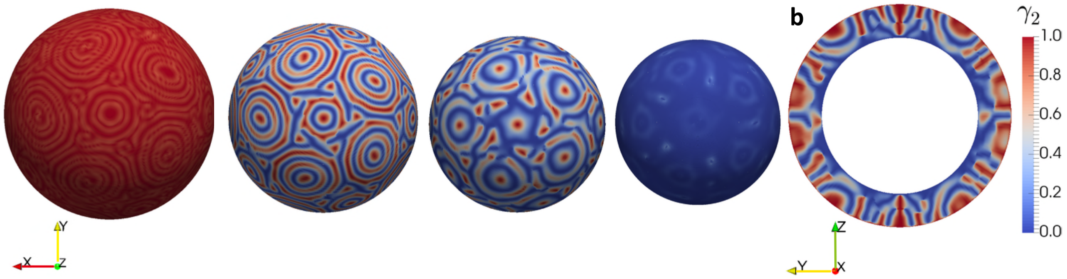

Supplementary Fig. 17: **Simulated configuration of symmetric cholesteric liquid crystal (CLC) shell with hybrid boundary conditions.** An equilibrium profile on a symmetric shell with normal boundary conditions on the outer surface and tangential boundary conditions on the inner surface. The model parameters are $t = -1.79$, $\xi_R = 1/50$, $\eta = 1$, $\sigma = 10\pi$, $\omega = \omega_1 = \omega_2 = 0.02$. The profile of $\gamma_2 = |\mathbf{n} \cdot \mathbf{e}_\xi|$ where $\mathbf{e}_\xi$ is the unit ξ-direction in bispherical coordinate, on (a) the surface with $r = 1$, 0.9, 0.8, 0.7 (from left to right), and (b) cross-section.

Supplementary References

1. Majumdar, A. Equilibrium order parameters of nematic liquid crystals in the Landau-de Gennes theory, *Eur. J. Appl. Math.* **2010**, *21*, 181–203

2. Han, Y. and Dalby, J. and Majumdar, A. and Carter, B. and Machon, T. Uniaxial versus biaxial pathways in one-dimensional cholesteric liquid crystals, *Phys. Rev. Res.*, *4*, L032018 (2022)

3. Rapini, A. and Papoular, M. Surface anchoring of nematic liquid crystals, *J. Phys. Coll.*, *30*, 54–66 (1969)

4. Noh, J., Wang, Y., Liang, H.-L., Jampani, V.S.R, Majumdar, A. and Lagerwall, J.P.F. Dynamic tuning of the director field in liquid crystal shells using block copolymers, *Phys. Rev. Res.*, *3*, 033160 (2020)

5. Gilbert, P.H. and Giacomin, A.J. Transport phenomena in bispherical coordinates, *Phys. Fluids*, *31*, 021208 (2019)

6. Nocedal, J. and Wright, S.J. Numerical Optimization, Springer (1999)

7. Cladis. P.E. and Torza, S. Growth of a smectic-A from a bent nematic phase and smectic light valve, *J. Appl. Phys.*, **46**, 584–599 (1975)

8. Liang, H.-L., Zentel, R., Rudquist, P. and Lagerwall, J.P.F. Towards tunable defect arrangements in smectic liquid crystal shells utilizing the nematic-smeectic transition in hybrid-aligned geometries. *Soft Matter*, *8*, 5443–5450 (2012)

9. Noh, J. and Lagerwall, J.P.F. Topological defect-guided regular stacking of focal conic domains in hybrid-aligned smectic liquid crystal shells. *Crystals*, *11*, 913 (2021)
