# Supplementary material for: Tunable templating of photonic microparticles via liquid crystal order-guided adsorption of amphiphilic polymers in emulsions
Source: Nat Commun. 2024 Feb 15;15:1404. doi: 10.1038/s41467-024-45674-5 (PMC10869789; doi:10.1038/s41467-024-45674-5)
Supplement: Supplementary file 3 — Description of Additional Supplementary Files [file 41467_2024_45674_MOESM3_ESM.pdf]

**Description of Additional Supplementary Files**

File Name: Supplementary Movie 1
Description: Transmission POM Movie of CLC base mixture shells stabilized by 87-89% hydrolyzed PVA heated from room temperature to above clearing transition, and start of re-cooling. 8x sped up. Fig. 2(a-h) corresponds to 0:04, 0:15, 0:29, 0:35, 0:42, 0:49, 0:55, 1:29 on this Movie.

File Name: Supplementary Movie 2
Description: Transmission POM Movie of 6% HDDA mixture shells stabilized by 87–89% hydrolyzed PVA heated from room temperature to above clearing transition, and start of re-cooling. 8x sped up. Fig. 6(a-l) 0:04, /, 0:10, 0:16, 0:31, 0:38, 0:46, 0:54, 1:12, 1:25,1:30, 1:35 on this Movie.

File Name: Supplementary Movie 3
Description: Transmission POM Movie of 6% HDDA mixture shells stabilized by 87-89% hydrolyzed PVA polymerized at different temperatures. 8x sped up. Fig. 3(a-c) corresponds to 0:04, 0:36, 2:09 on this Movie.

File Name: Supplementary Movie 4
Description: Reflection POM Movie of 6% HDDA mixture shells stabilized by 87-89% hydrolyzed PVA with glycerol-rich isotropic phases heated from room temperature to above clearing transition, and start of re-cooling. 8x sped up. Fig. 4(a-h) corresponds to 0:04, 0:17, 0:27, 0:44, 0:55, 1:21, 1:32, 2:50 on this Movie.

File Name: Supplementary Movie 5
Description: Transmission POM Movie of CLC base mixture shells stabilized by F-127 heated from room temperature to above clearing transition, and start of re-cooling. 8x sped up. Fig. 10(a-l) corresponds to 0:05, 0:10, 0:14, 0:17, 0:28, 0:36, 0:47, 0:53, 1:33, 1:45, 1:47, 1:52 on this Movie.

File Name: Supplementary Movie 6
Description: Transmission POM Movie (side view) of 0% HDDA mixture shells stabilized by F-127 slowly heated from room temperature to 63◦C. The panels in Fig. 11 are still frames from this Movie, with time stamps (a-n): 0:02, 0:05, 0:10, 0:14, 0:20, 0:26, 0:32, 0:38, 0:44, 0:49, 0:54, 1:00, 1:05, 1:11.

File Name: Supplementary Movie 7
Description: Transmission POM Movie of 6% HDDA mixture shells stabilized by 99+% hydrolyzed PVA heated from room temperature to above clearing transition, and re-cooling. 8x sped up. Fig. 7(a-h) correspond to 0:14, 0:17, 0:18, 0:19, 1:15, 1:18, 1:30, 1:42 on this Movie.

File Name: Supplementary Movie 8
Description: Transmission POM Movie of 6% HDDA mixture shells stabilized by 87-89% hydrolyzed PVA cooled from isotropic until shell rupture. 8x sped up.
